# Supramolecular Assemblies Showing Thermally Activated Delayed Fluorescence

**DOI:** 10.1002/smsc.202100022

**Published:** 2021-06-04

**Authors:** Thomas A. Comerford, Eli Zysman-Colman

**Affiliations:** ^1^ Organic Semiconductor Centre EaSTCHEM School of Chemistry University of St Andrews St Andrews KY16 9ST UK

**Keywords:** fluorescence, supramolecular assemblies, thermally activated delayed fluorescence

## Abstract

Supramolecular assemblies based on luminescent components offer significant advantages over their discrete counterparts, including improved quantum yields, stability, and tunability. Following interest as advanced optoelectronic materials, thermally activated delayed fluorescence (TADF) emitters are incorporated into a range of supramolecular structures. Herein, a summary of the known examples of emissive supramolecular systems that operate via a TADF mechanism with comparisons, where possible, with their discrete counterparts is presented. While the types of supramolecular structures are diverse, there are limited examples shown for each class. With the increase in photophysical performance and/or emergence of new photochemical properties upon going from molecular to supramolecular, the potential that these systems hold becomes apparent.

## Graphical abstract

Thermally activated delayed fluorescence (TADF) emitters are increasingly used in advanced optoelectronic materials, though they remain underexploited in supramolecular systems. In evolving from molecular to supramolecular systems, new, emergent properties are seen as well as enhancement of the photophysical properties. Herein, known examples of supramolecular TADF are summarized and compared, where possible, with their discrete molecular counterparts.

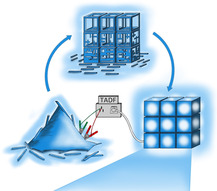

## 1 Introduction

**Figure 1:**
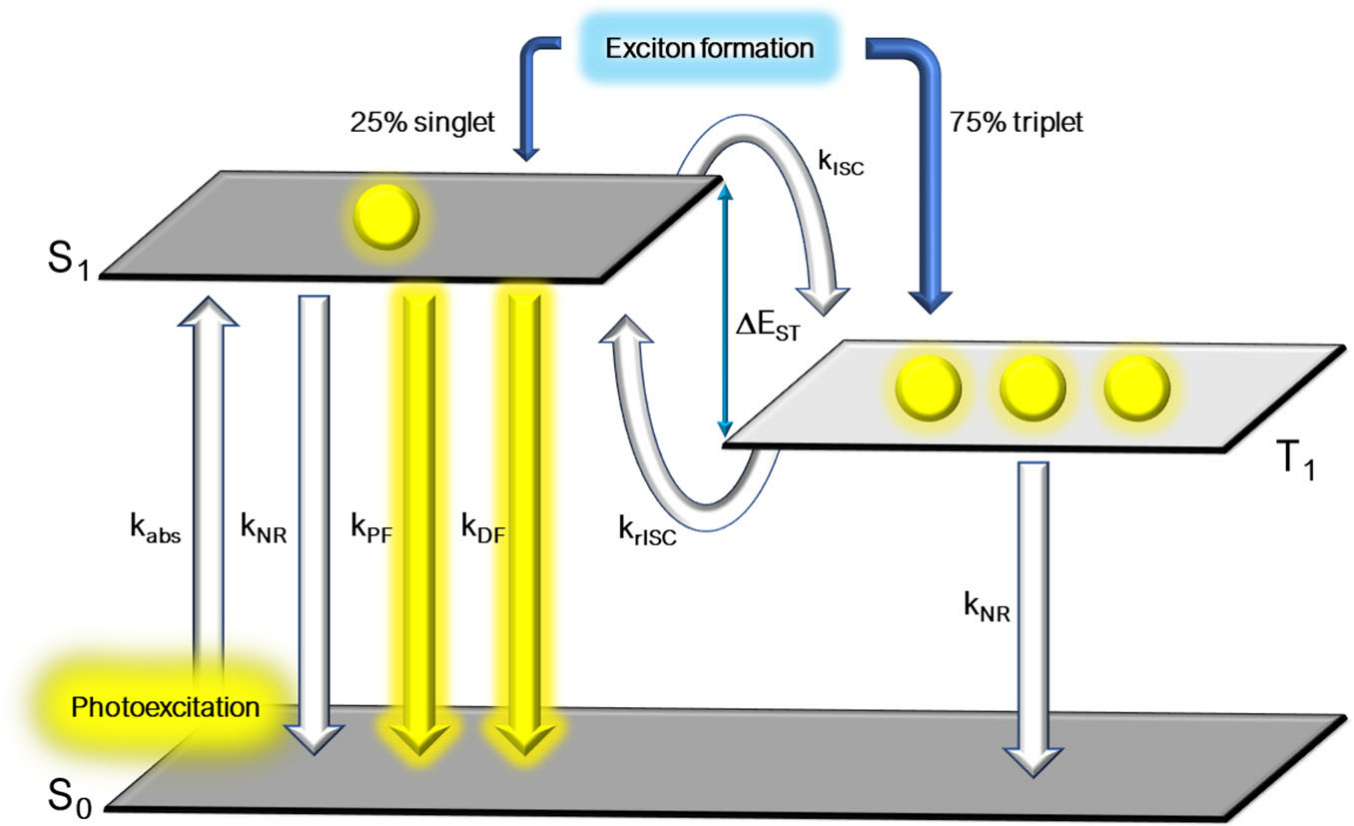
TADF mechanism involving organic emitters: *k*_abs_ = rate constant of absorption, *k*_NR_ = rate constant of nonradiative decay, *k*_F_ = rate constant of fluorescence, *k*_DF_ = rate constant of delayed fluorescence, *k*_ISC_ = rate constant of ISC, and *k*_rISC_ = rate constant of rISC.

Thermally activated delayed fluorescence (TADF) materials have attracted considerable interest due to their strong potential in replacing phosphorescent complexes as emitters in state‐of‐the‐art organic light‐emitting diodes (OLEDs).^[^ ^1^, ^2^, ^3^, ^4^, ^5^, ^6^ ^]^ The ability of organic TADF materials to harness singlet and triplet excitons, the latter via reverse intersystem crossing (rISC), to achieve up to 100% internal quantum efficiency in the device is key to the attention these advanced optoelectronic emitters have received. High‐efficiency blue and deep‐blue OLEDs have been recently reported with external quantum efficiencies of 25.7% and 28.2% respectively, overcoming the 25% spin‐statistical bottleneck for these emitters by harvesting triplet electrons via rISC.^[^ ^7^ ^]^ TADF compounds are also increasingly being used as photocatalysts (**Figure** **1**).^[^ ^8^ ^]^ The presence of triplet states in the TADF mechanism makes these systems useful as sensors for oxygen,^[^ ^9^, ^10^ ^]^ and the temperature‐dependent nature of TADF emission allows for their use as molecular thermometers.^[^ ^10^, ^11^, ^12^, ^13^ ^]^ The long excited‐state lifetimes endemic to TADF emitters coupled with their lower‐toxicity profiles than organometallic complexes have been factors driving their exploration as bioimaging reagents.^[^ ^14^, ^15^, ^16^ ^]^ In particular, their long lifetimes permit a clear distinction between the TADF marker and short‐lived emission from biological autofluorescence.^[^ ^17^ ^]^ As the use of these materials increases and the scope of their use diversifies, so does the need to achieve greater control of their properties. Incorporation of TADF materials into supramolecular assemblies, unlike other classes of luminescent compounds, is underexplored and may fundamentally change their properties via intra/intermolecular interactions, reducing unwanted nonradiative decay and giving new reactivity when used in photocatalysis. In this Review, we focus on examples where TADF occurs from organic units within supramolecular systems. There are examples of supramolecular organometallic complexes that display TADF, for example, the Cu(I)‐containing structures reported by Lescop and coworkers^[^ ^18^, ^19^, ^20^ ^]^; however, these are outside the scope of this Review.

## 2 Supramolecular TADF: A Diverse Lineup

### 2.1 Supramolecular TADF Photocatalysis

**Figure 2:**
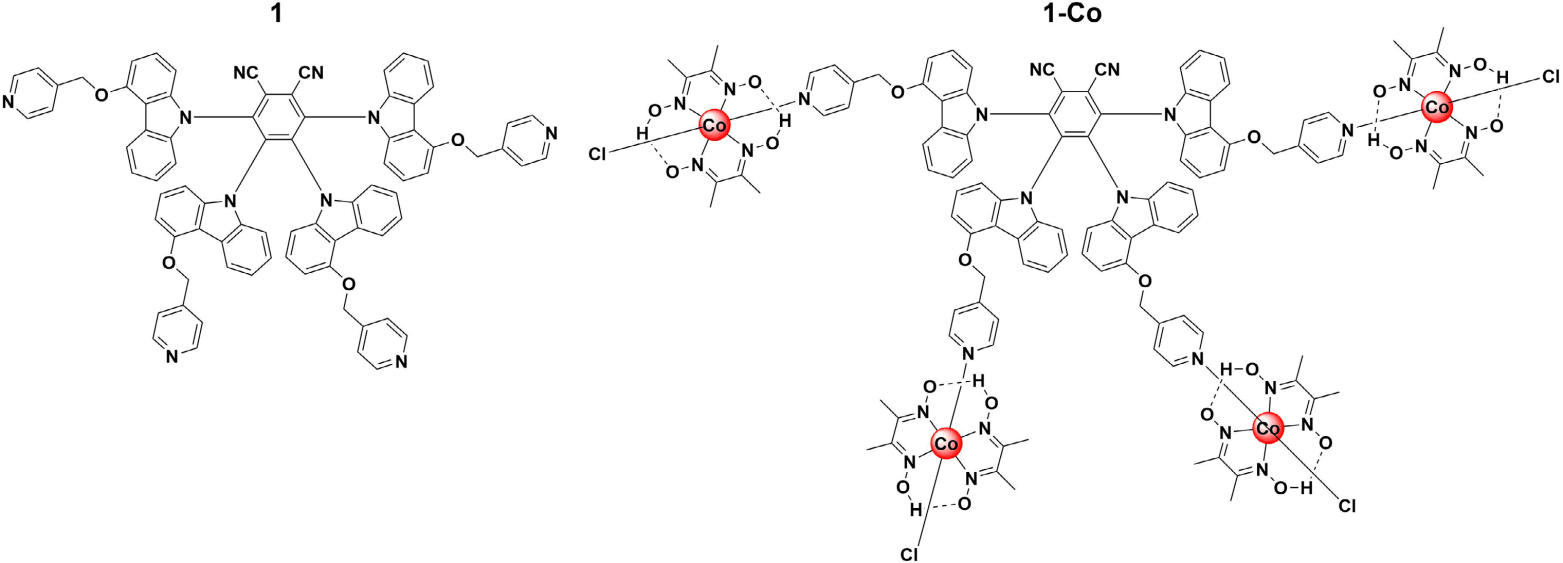
Precursor TADF compound **1**, and supramolecular assembly **1‐Co**, following coordination of four cobaloxime species to the pyridine groups of **1**. Structure of **Co1** reproduced with permission.^[^ ^33^ ^]^ Copyright 2019, Royal Society of Chemistry.

**Figure 3:**
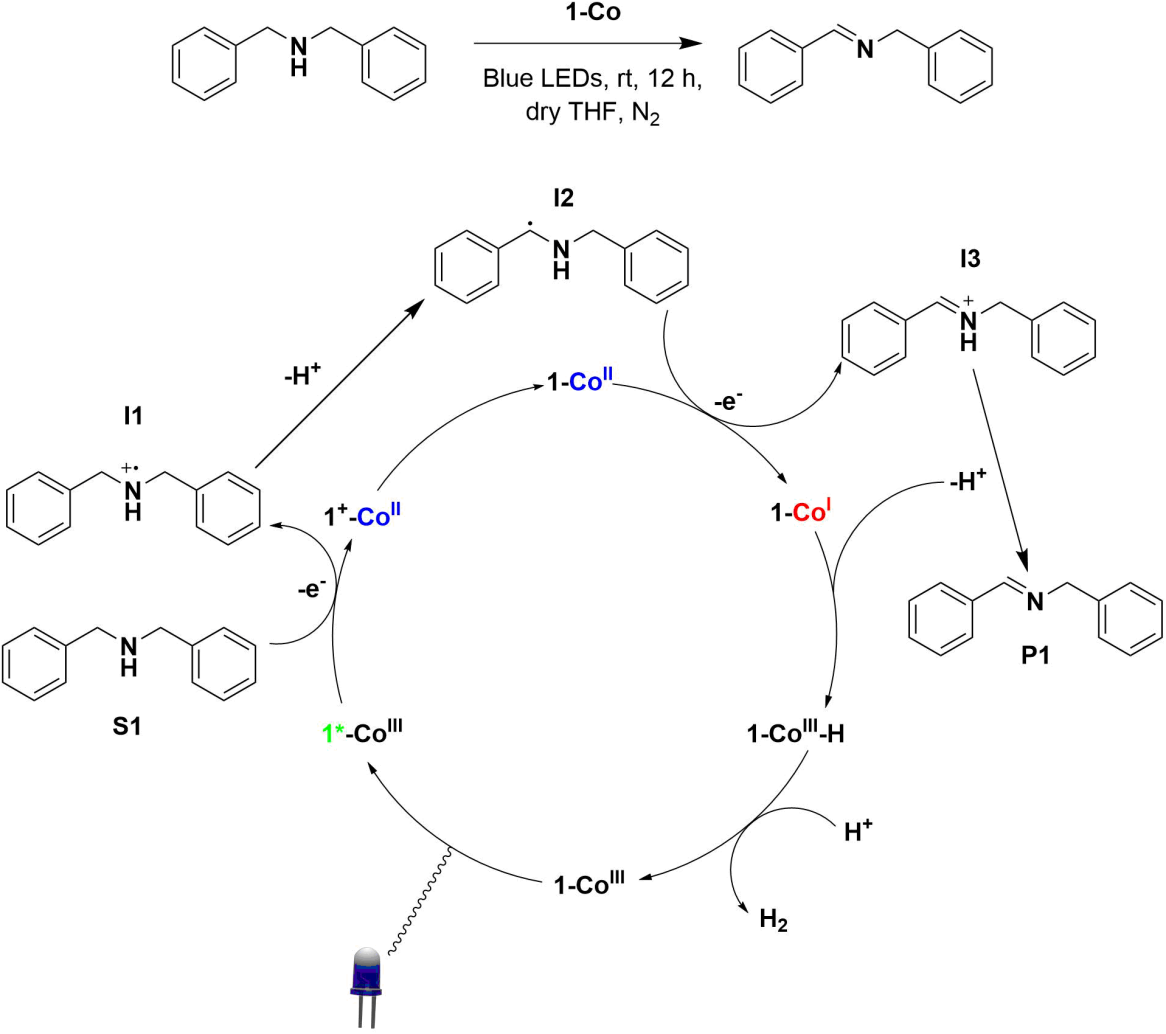
Photocatalytic cycle for the CAD of dibenzylamine to give hydrogen gas. Reproduced with permission.^[^ ^33^ ^]^ Copyright 2019, Royal Society of Chemistry.

A subclass of photocatalysts comprising a photosensitizer linked to a catalyst, the so‐called chromophore‐catalyst assembly, have been used as hydrogen‐evolving/CO_2_ reduction cooperative catalyst systems.^[^ ^21^, ^22^, ^23^ ^]^ The photosensitizing units undergo excited‐state electron transfer to the appended catalysts, with common photosensitizers being Ir(III),^[^ ^24^, ^25^, ^26^ ^]^ Ru(II),^[^ ^27^, ^28^, ^29^ ^]^ with the use of organic species such as BODIPY dyes,^[^ ^30^, ^31^ ^]^ and fluorescein also known.^[^ ^32^ ^]^ Chao and Zhao reported a TADF chromophore comprising a 1,2‐dicyanobenzene core with four methoxy pyridincarbazole groups, 1.^[^ ^33^ ^]^ This species can coordinate to four cobalt(III) chloro(pyridine)cobaloxime species to afford the supramolecular assembly, 1‐Co, which they showed engaged in catalytic acceptor‐less dehydrogenation (CAD) (**Figure** **2**). CAD involves the two‐electron oxidation of organic precursors to give oxidized products and two equivalents of hydrogen, allowing for the generation of hydrogen gas from organic species without the need for stoichiometric inorganic oxidants. CAD is an attractive route for green hydrogen production.^[^ ^34^ ^]^ The emission spectrum of 1 in DCM is broad and featureless, characteristic of emission from a charge‐transfer (CT) state, and is centered at λ_PL_ = 591 nm; the compound likewise shows positive solvatochromism that is associated with CT excited states. The photoluminescence quantum yield, Φ_PL_, is only 7.2%. Density functional theory (DFT) calculations confirmed the donor−acceptor nature of 1, showing a highest occupied molecular orbital (HOMO) that is largely distributed over the carbazole moieties with the lowest unnocupied molecular orbital (LUMO) localized on the electron‐poor phthalonitrile core. The ΔE_ST_ value for 1 is small, quoted at 0.13 eV, suggesting that TADF is plausible.^[^ ^35^ ^]^ With the measured photoluminescence lifetime in degassed CH_2_Cl_2_ at 298 K found to be *τ*_PL_ = 17.4 μs, the excited state of 1 is sufficiently long to allow for its engagement in photocatalytic processes. Compared with 1, 1‐Co had a lower photoluminescence quantum yield and decreased lifetime (Φ_PL_ = 2.9%, *τ*_PL_ = 13.8 μs), both attributed to photoinduced electron transfer (PET) from 1 to the cobalt centers. Investigation of the redox potentials of 1‐Co showed electronic separation in the ground state between the cobalt centers and the organic core, with distinct irreversible redox waves seen for Co^III^/Co^II−−^ and 1^+^/1 redox couples at −0.63 and +1.38 V versus saturated calomel electrode in acetonitrile, respectively. Following confirmation of electron transfer from 1 to the cobaloxime groups, the assembly was used in the CAD of secondary amines. Irradiation with blue LEDs (450 ± 10 nm, 3 W) of a degassed, dry THF solution of dibenzylamine, S1, with 0.04% loading of 1‐Co at room temperature gave a turnover number (TON) of 305 for hydrogen production after 12 h. In contrast, using a mixture of cobaloxime and 1 in solution afforded a significantly lower TON of 53. This increase in efficiency for the supramolecular system over the mixture of catalysts is attributed to more efficient electron transfer between the two components as a result of the covalent bond between them. A control experiment without irradiation was conducted and showed no H_2_ production, demonstrating the photochemical nature of this reaction. The imine product, P1, of the CAD of S1 was observed by ^1^H NMR, which provided evidence that S1 was the source of H_2_. The highest TON achieved was 895 when using 1,2,3,4‐tetrahydroisoquinoline as the substrate and with a 0.04% catalyst loading of 1‐Co. PET was confirmed by in situ monitoring of the UV−vis absorption spectra, which showed the emergence of a Co^II^ species arising from electron transfer from 1* to Co^III^; there was also an increase in absorption between 550 and 700 nm, attributed to the formation of a Co^I^ species.^[^ ^36^ ^]^ The proposed mechanism is shown in **Figure** **3**. Upon PET from 1*‐Co^III^, 1^+^‐Co^II^ is formed. Oxidized 1^+^ can then oxidize S1 to give a radical cation, I1, which then undergoes hydrogen abstraction to give a strongly reducing radical species I2 followed by oxidation to the iminium ion I3 yielding Co^I^. Stephenson and coworkers demonstrated the nucleophilic addition to and oxidative alkylation of photogenerated iminium species to give a wide range of products, making this intermediate valuable in organic synthesis.^[^ ^37^, ^38^, ^39^ ^]^ Deprotonation of I3 gives the product P1. Co^I^ is protonated by I3 to give 1‐Co^III^−H and P1. Finally, 1‐Co^III^−H is protonated a final time to regenerate 1‐Co^III^ and release H_2_. The authors note that Co^II−−^ cannot be reduced by 1* due to the less negative potential of E(1^+^/1*) versus E(Co^II^/Co^I−−^) (−0.71 vs −1.12 V), supporting their proposed mechanism; however, other plausible pathways exist such as homolysis of Co^III^−H.^[^ ^40^ ^]^

### 2.2 Supramolecular Gels

**Figure 4:**
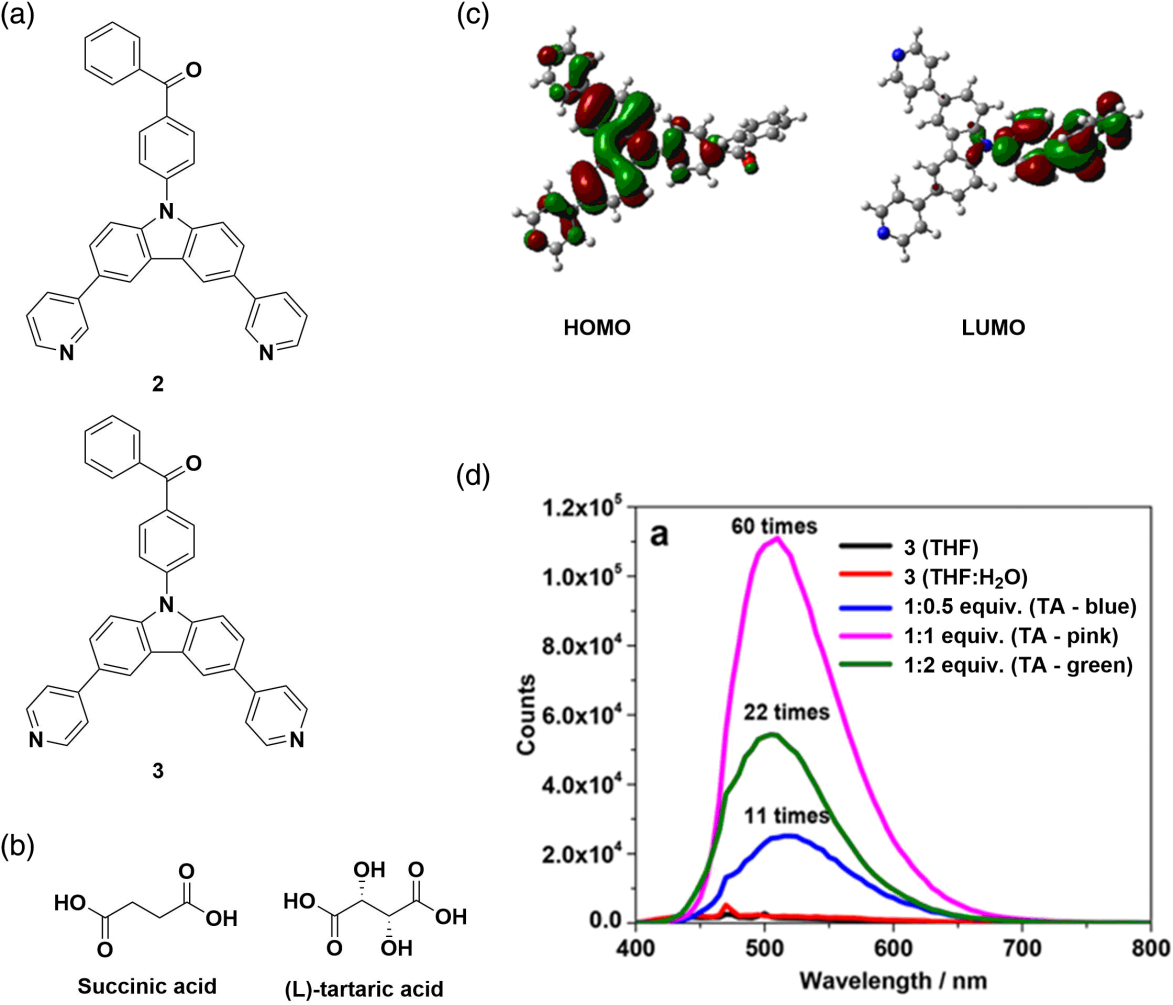
a) Structures of TADF emitters 2 and 3. b) Structures of succinic acid and (L)‐tartaric acid. c) HOMO and LUMO of 3. d) Increase in emission intensity with increased strength of the gel by modulation of the ratio of (L)‐tartaric acid:**3**. c,d) Reproduced with permission.^[^ ^41^ ^]^ Copyright 2018, American Chemical Society.

Zysman‐Colman and coworkers reported the first example of a TADF gel formed from a carbazole−benzophenone TADF acceptor, 3.^[^ ^41^ ^]^ These were formed from the mixture of a TADF emitter 3 (**Figure** **4**a) with diacids (Figure 4b); the structurally related compound 2 did not form any gel when mixed with either succinic or tartaric acid. DCM solutions of 2 and 3 showed broad absorption bands at 356 and 326 nm, respectively, attributed to intramolecular charge transfer (ICT) from carbazole to benzophenone that was corroborated by DFT calculations (Figure 4c). The emission maxima of 3 in degassed DCM and in PMMA‐doped films (10 wt%) at 298 K are 477 and 449 nm with associated quantum yields under N_2_ measured at 52% and 21%, respectively. These quantum yields decreased significantly to Φ_PL_ = 10% and 17%, respectively, in the presence of O_2_, confirming that triplet states are involved in the emission of this compound. A yellow/green gel formed with enhanced emission at *λ*_PL_ = 500 nm, when one equivalent of succinic acid was added to a solution of 3. This gel was weak and flowed when the vial was inverted, with a critical gel concentration (CGC) of 5 mg mL^−1^. When using L‐tartaric acid, the viscosity increased markedly, showing that a stronger gel formed with a CGC of 3 mg mL^−1^. This was attributed to the increased hydrogen bonding possible with (L)‐tartaric acid. The emission maximum for the (L)‐tartaric acid gel was red‐shifted compared with the succinic acid‐containing gel at *λ*_PL_ = 510 nm. The emission intensity is dependent on the amount of L‐tartaric acid, increasing by 11‐fold with 0.5 equivalent and 60‐fold with 1 equivalent of diacid compared with free 3. However, using more than 1 equivalent of diacid disrupted the gel and decreased the emission intensity (Figure 4d). The authors propose that H bonding between pyridine of 3 and acid units is responsible for formation of the gel, confirmed by fourier transform infrared spectroscopy. Transient photoluminescence measurements revealed that the 1:1 3:(L)‐tartaric acid gel possessed biexponential decay kinetics consistent with the gel being TADF, with lifetimes *τ*_PL_ = 20 ns and 2.3 μs, representing the prompt and delayed emission, respectively. The gel fibers were shown to be amorphous, with no change in the structure shown under high vacuum. The photoluminescence quantum yield of the xerogel is six times greater that of the neat film (Φ_PL_ = 36% vs 6%). As the fibers were not damaged by high vacuum and retained their structure, these gels have a potential application in OLEDs.

### 2.3 TADF Supramolecular Metallocages

**Figure 5:**
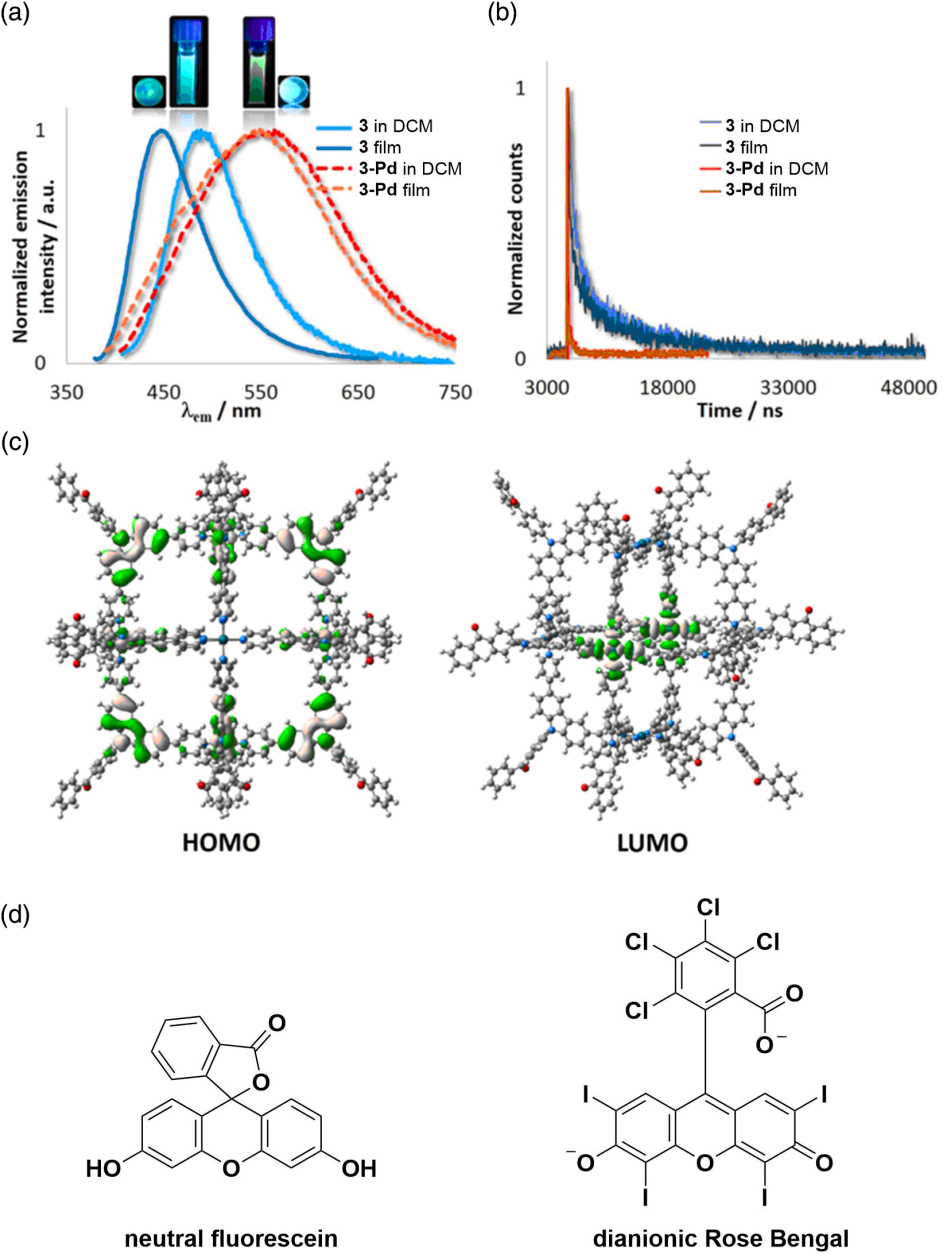
a) Steady‐state emission spectra of 3 and 3‐Pd in DCM solution and 10 wt% PMMA‐doped thin films. b) Transient photoluminescence (PL) decays of 3 and 3‐Pd in DCM and thin films showing the quenching of delayed emission upon complexation of 3 to Pd(II). c) HOMO and LUMO plots for 3‐Pd showing LUMO distribution over the Pd(II) vertices. d) Structures of the two xanthene dyes, fluorescein and rose bengal. a–c) Reproduced with permission.^[^ ^45^ ^]^ Copyright 2018, American Chemical Society.

Supramolecular metallocages comprise metallic vertices linked by organic bridging ligands, encompassing a wide range of shapes and sizes depending on the coordination geometry of the linker and the choice of metal.^[^ ^42^ ^]^ Luminescent metallocages may be emissive due to their metallic ions, with the most common examples being cages based on Ir(III) and Ru(II),^[^ ^43^ ^]^ or through fluorescent organic linkers as such as those seen from Stang and coworkers with aggregation‐induced emission (AIE) from tetraphenylethylene linkers.^[^ ^44^ ^]^ Zysman‐Colman et al. explored the use of **3** in the construction of a supramolecular metallocage using Pd(II) ions as vertices. The cage could encapsulate organic dye molecules. Depending on the nature of the dye, either energy (PEnT) or electron (PeT) transfer to encapsulated guests could be observed.^[^ ^45^ ^]^ As 3 has a vector angle between pyridine rings of 93.5°, it is an appropriate ligand to make M_6_L_12_ cages, as evidenced by the work of Fujita and coworkers with ligands having similar vector angles of around 90°.^[^ ^46^ ^]^ DFT modeling of the metallocage 3‐Pd (**Figure** **5**c) predicted cubic structure with an internal volume of 6400 Å. The emission spectrum of 3‐Pd in degassed DCM was red‐shifted and broader compared with that of 3 and with a significantly lower photoluminescence quantum yield (3; *λ*_max_ = 477 nm, Φ_PL_ = 52%, 3‐Pd; *λ*_max_ = 555 nm, Φ_PL_ = 4% in DCM). While 3 shows triexponential photoluminescence decay with a prompt fluorescence, *τ*_PL_ = 15.1 ns, and biexponential delayed fluorescence, *τ*_PL_ = 0.69 and 9.80 μs, components in degassed DCM, 3‐Pd shows no delayed component, with a biexponential lifetime of *τ*_PL_ = 3 and 30 ns. DFT modeling of 3‐Pd revealed that while the HOMO remains largely centered on the carbazole donor moieties, the LUMO is no longer distributed over the benzophenone acceptor and is instead centered on the Pd(II) and the coordinated pyridine ligands. The implication of an ligand‐to‐metal charge‐transfer state to formally antibonding palladium *d*‐orbitals is the origin of the significant quenching of the emission in the metallocage. The 3‐Pd cage was used to encapsulate two xanthene dyes: fluorescein (F) and rose bengal (RB) (Figure 5d). Analysis of the host−guest complexes via electrospray ionization mass spectrometry (ESI‐MS) showed that up to three molecules of neutral F and up to two molecules of RB in its quinoid dianionic form could be encapsulated. Upon addition of a solution of 3‐Pd to a solution of F or RB, there were marked changes in the UV−vis absorption spectra of the dyes. Addition of 3‐Pd (from 0.1 to 3.0 equiv.) to a solution of F resulted in the reduction of the absorption band at 522 nm of the open quinoid form, eventually producing a colorless solution, which suggests that the cavity of the 3‐Pd favors the neutral form of F. No change in the absorption spectrum of RB was observed upon addition of 3‐Pd (from 0.1 to 3.0 equiv.) to a solution of RB, thus confirming the presence of the open dianion quinoid form of RB, as corroborated by ESI‐MS. For the 3‐Pd⊂F host−guest complex, PET was observed, resulting in the formation of [F]^.+^[3‐Pd]^−^, with complete quenching of the emission of both F and 3‐Pd. In contrast, when 3‐Pd was added to a DMSO solution of RB, photoinduced energy transfer was observed, with gradual quenching of the green emission from 3‐Pd and a concomitant enhancement of the orange emission from RB at 582 nm. Stern−Volmer quenching analysis of the host−guest complex showed efficient photoinduced energy transfer, with a Stern−Volmer constant of *K*_SV_ = 1.22 × 10^4^ M^−1^ and a quenching rate constant of *k*_q_ = 4.07 × 10^11^ M^−1^ s^−1^. Förster energy transfer was proposed as the energy transfer mechanism.

### 2.4 Mechanically Interlocked Rotaxane⊂Emitter Species

**Figure 6:**
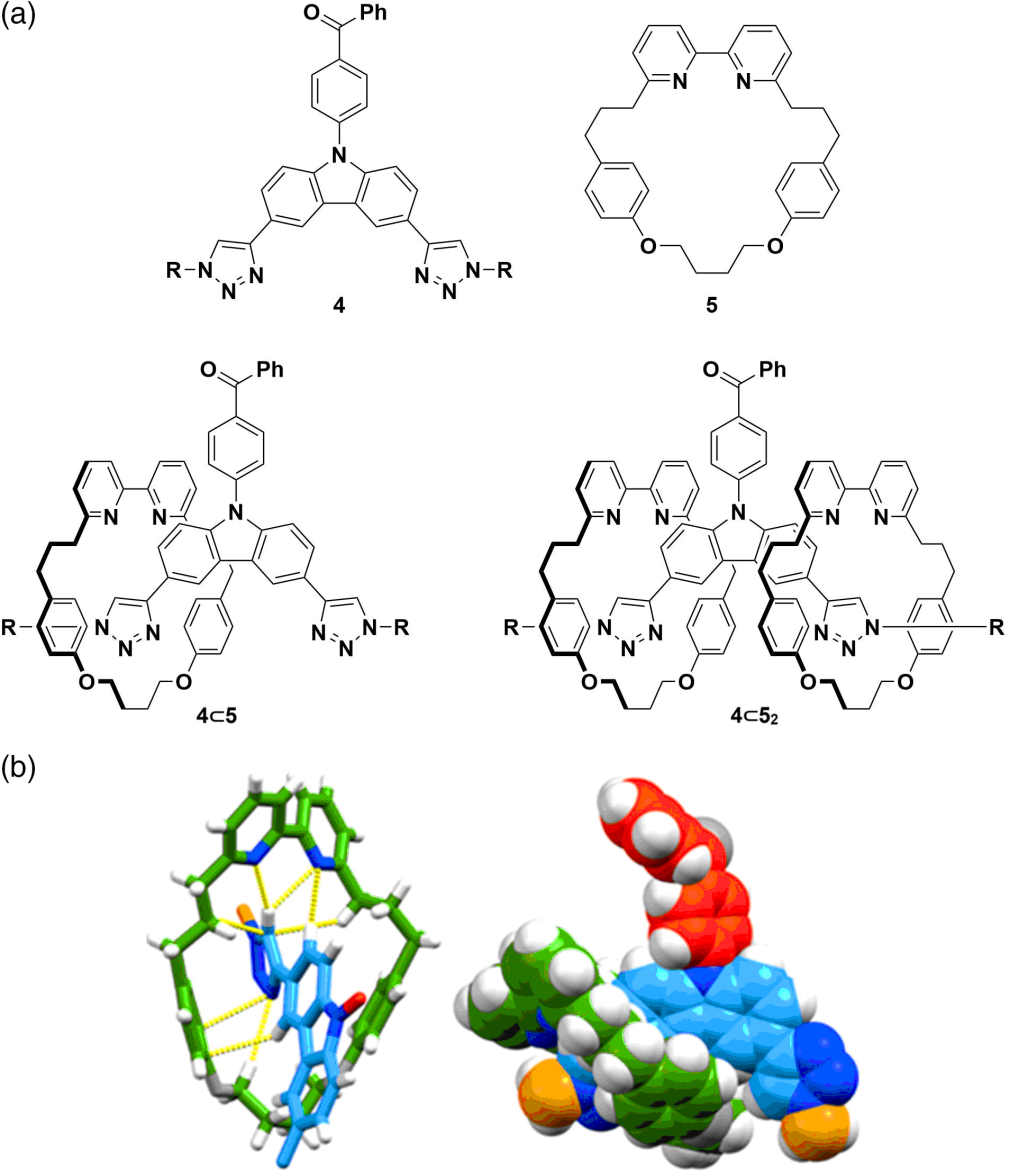
a) Structures of emitter 4 and macrocycle 5, along with host⊂guest systems 4⊂5 and 4⊂5_2_. b) Close‐contact interactions between the bipyridine nitrogen atoms of 5 and the C−H groups on the triazole group in 4⊂5 combined with other intramolecular interactions and a space filling model of 4⊂5. Reproduced under the terms of the CC‐BY 4.0 license.^[^ ^48^ ^]^ Copyright 2021, The Authors, published by Wiley‐VCH.

Rotaxanes are a class of mechanically interlocked molecules, typically consisting of a linear guest species and a cyclic host species held together by noncovalent forces.^[^ ^47^ ^]^ Recently, Zysman‐Colman, Goldup, Penfold, et al. reported the fine modulation of photophysical properties of a carbazole−benzophenone‐based TADF emitter 4, a compound similar to 3 but with triazole groups in place of pyridine, upon interaction with macrocycle 5 to give rotaxane systems 4⊂5 and 4⊂5_2_ (**Figure** **6**a).^[^ ^48^ ^]^^1^H NMR and single‐crystal X‐ray diffraction analyses showed the triazole protons to be hydrogen bonded to the macrocycle bipyridine moiety (Figure 6b). Photophysical measurements in toluene probed the effect of mechanical bond formation on the optoelectronic properties. Complexation to the macrocycles led to an increase in the photoluminescence quantum yield under N_2_ and versus the uncomplexed emitter (4; Φ_PL_ = 11% 4⊂5; Φ_PL_ = 31%, 4⊂5_2_; Φ_PL_ = 30%) along with a decrease in the Δ*E*_ST_; in addition, there was an observed increase in the photostability of these systems under UV irradiation. DFT calculations of these systems show that while the benzophenone‐centered LUMO remains largely unchanged upon complexation in 4⊂5 and 4⊂5_2_, the HOMO is destabilized due to hydrogen bonding between the C—H bond on the triazole arms and the bipyridine nitrogen atoms of 5. In summary, the mechanical bond formed between 4 and 5 allowed for fine tuning of the emission, a decrease in the Δ*E*_ST_, an increase in photoluminescence quantum yields, and greater photostability, all traits that are desirable when designing emitters, particularly for electroluminescent devices.

### 2.5 TADF Metal−Organic Frameworks (MOFs)

**Figure 7:**
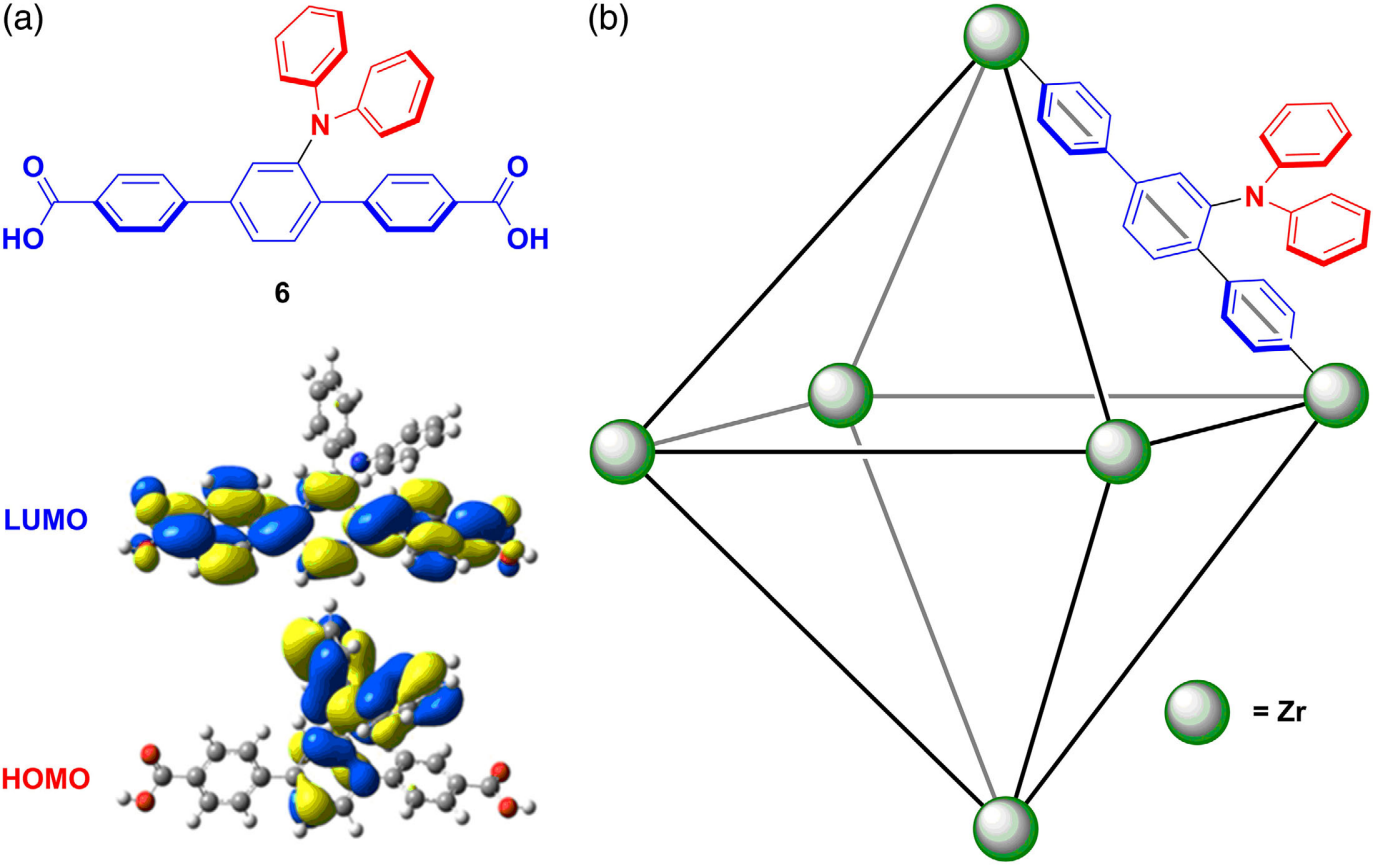
a) Structure of 6 with calculated HOMO and LUMO plots. b) Structure of MOF‐1 with 6 binding via carboxylic acid groups to zirconium centres. Adapted with permission.^[^ ^70^ ^]^ Copyright 2018, Royal Society of Chemistry.

Metal−organic frameworks (MOFs) are extended supramolecular systems comprising metal ions or clusters held together with organic linkers via coordination bonds.^[^ ^49^ ^]^ Their porous nature combined with the near‐limitless diversity of the structure associated with different combinations of metals and organic linkers has led to significant interest in their exploitation in numerous applications, including gas storage and separation^[^ ^50^, ^51^, ^52^ ^]^ and catalysis.^[^ ^53^, ^54^ ^]^ A large subset of MOFs are luminescent and these have been used in sensing,^[^ ^55^, ^56^, ^57^, ^58^ ^]^ bioimaging,^[^ ^48^, ^49^, ^59^, ^60^ ^]^ light‐emitting devices,^[^ ^61^, ^62^, ^63^, ^64^ ^]^ and as photocatalysts.^[^ ^65^, ^66^, ^67^, ^68^, ^69^ ^]^ Adachi et al. reported the first example of a TADF MOF. The MOF links a TADF emitter, 6, containing two carboxylate groups with zirconium‐based vertices (**Figure** **7**).^[^ ^70^ ^]^ The linker 6 was chosen due to its small calculated Δ*E*_ST_ of 0.2 eV. For 6, there is a broad CT emission at 481 nm. The Δ*E*_ST_ of 6 was estimated to be 0.24 eV, in line with the calculations. Emission of 6 in 2 wt% PMMA‐doped films showed a biexponential decay, with prompt and delayed lifetimes, respectively, of 18 ns and 199 ms, and a Φ_PL_ of 39%, which decreased to 32% in the presence of air. The photophysical properties of MOF‐1 were measured following activation of the MOF‐1 at 400 K under vacuum. Compared with 6, the emission of MOF‐1 is red‐shifted and broader, centered at around 501 nm (Commission internationale de l'éclairage (CIE); 0.16, 0.18), with a slightly reduced Φ_PL_ = 30%; the Φ_PL_ decreased to 18% in the presence of air. The transient PL under N_2_ showed biexponential decay with lifetimes of 17 ns and 180 μs, representing the prompt and delayed emission, respectively. The reduction in photoluminescence quantum yield in air coincides with the loss of the delayed component of emission due to the quenching of the triplet state via oxygen. The decrease in the lifetime and red‐shifting upon MOF formation were suggested to arise from coordination to the electron‐poor zirconium centers.

**Figure 8:**
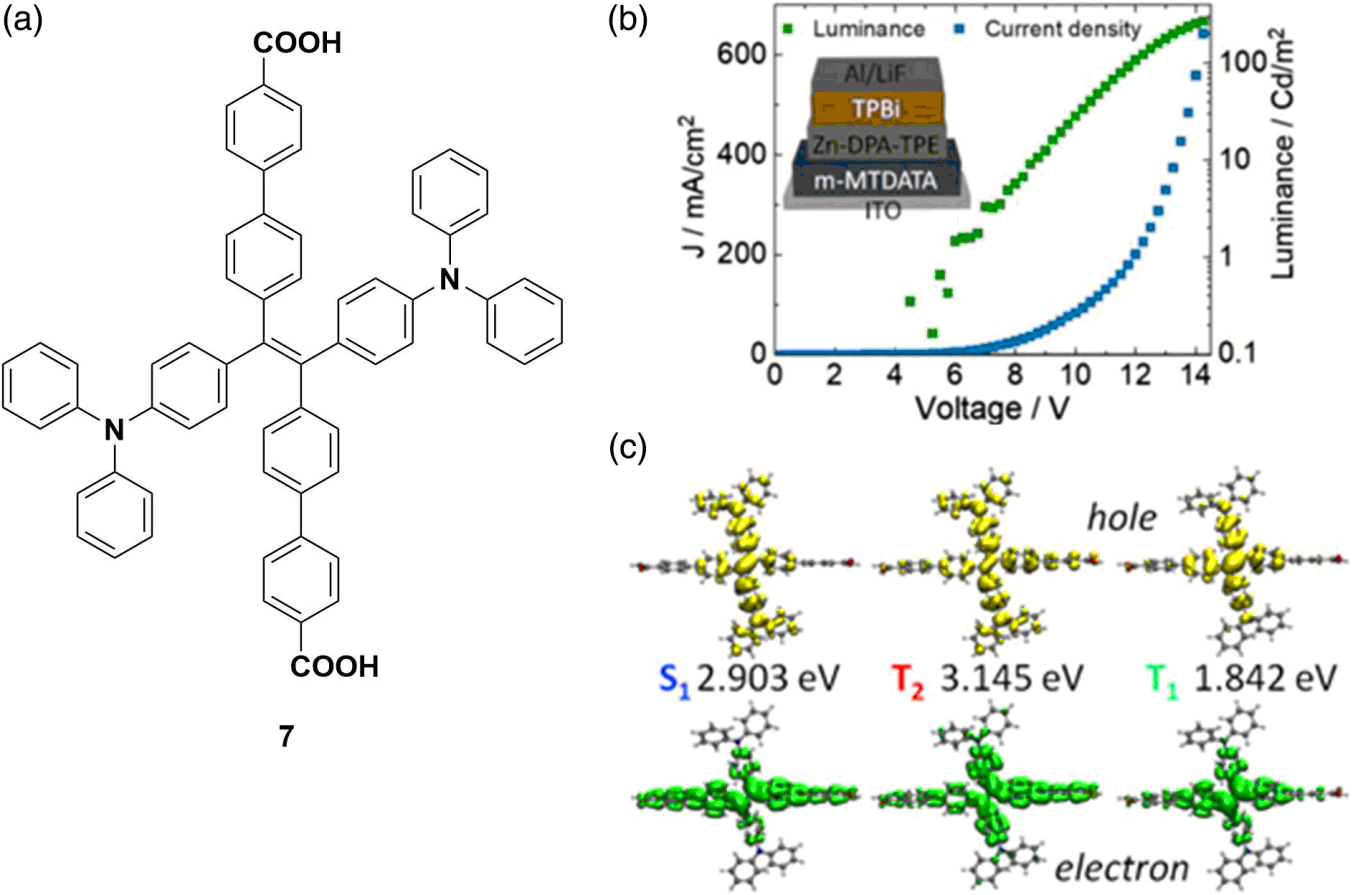
a) Structure of 7. b) Structure of OLED (m‐MTDATA = 4,4′,4″‐tris[phenyl(m‐tolyl)amino]triphenylamine, TPBi = 2,2′,2″‐(1,3,5‐benzinetriyl)‐tris(1‐phenyl‐1‐H‐benzimidazole) and the variance of luminance and current of the OLED with voltage. c) TD‐DFT‐calculated hole and electron distributions and energies of 7 within MOF‐2. b,c) Reproduced with permission.^[^ ^71^ ^]^ Copyright 2020, Wiley‐VCH.

Haldar and Wöll et al. reported the formation of MOFs from a diphenylamine−tetraphenylethylene TADF emitter, 7 (**Figure** **8**a), which, upon incorporation into oriented thin films, displayed green electroluminescence.^[^ ^71^ ^]^ By orienting emitters within a film to give channels for charge carriers, the conductivity of a system can be improved to be closer to that of a single crystal.^[^ ^72^ ^]^ The formation of the MOF from 7 allows for a high concentration of emitters. The presence of the tetraphenylethene moiety, a well‐known AIE building block, ensures that no concentration quenching is observed in the MOF. Dilute ethanol solutions of 7 are poorly emissive, attributed to nonradiative decay, resulting from the free rotation of the phenyl groups, with a lifetime of 1.5 ns. Powder samples of 7, however, showed delayed fluorescence (*τ*_PL_ = ≈200 μs). Upon forming a crystalline, orientated, thin‐film MOF with zinc ions (MOF‐2), the same delayed component is seen but with a significantly higher emission intensity. The TADF nature of this emission was demonstrated by variable temperature PL studies that showed an increase in intensity of emission for MOF‐2 with increasing temperature, which is linked to the thermal upconversion of triplet excitons to form radiative singlets. The Φ_PL_ of MOF‐2 is 14%. The thin‐film MOF was integrated into an OLED, the composition of which is shown in Figure 8b. The device showed a high turn‐on voltage of 5.8 eV, demonstrating a rather low luminance of 270 cd m^−2^ at 14 V. Time‐dependent density functional theory (TD‐DFT) calculations provided support for the nature of TADF emission from MOF‐2. The authors propose that a hot‐exciton mechanism is the source of TADF, whereby an exciton forms at the T_2_ state and undergoes RISC to the S_1_ state; this is possible due to the small energy gap between T_2_ and S_1_ compared with the much larger energy gap between T_2_ and T_1_.^[^ ^73^ ^]^ Incorporation of 7 as a component of the crystalline MOF‐2 results in decreased molecular vibrations, thus giving rise to high ISC and rISC rates of 6.94 × 10^6^ s^−1^ and 1.91 × 10^7^ s^−1^, respectively, at 300 K.

### 2.6 TADF Metallocycles

**Figure 9:**
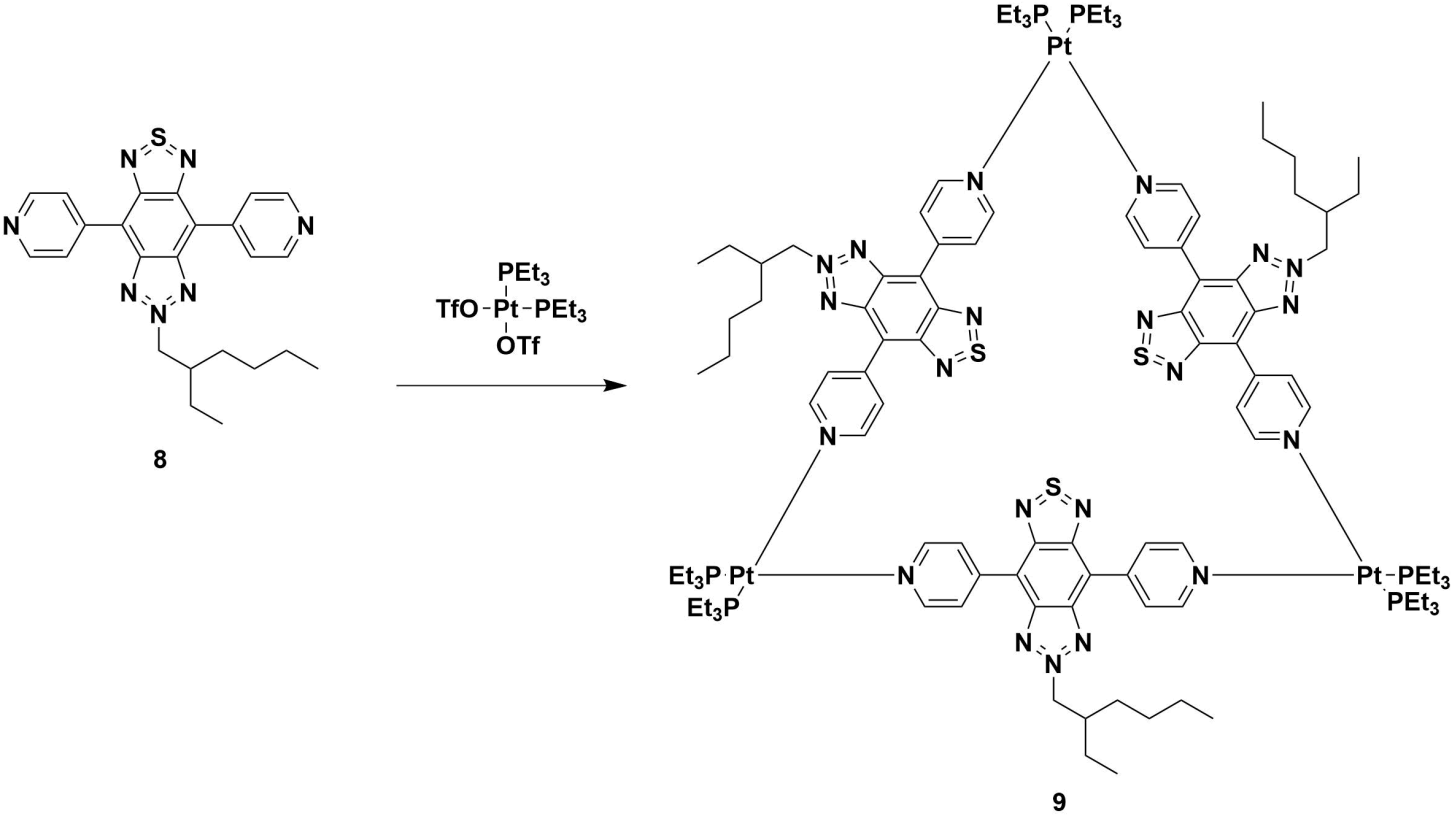
Novel organic linker 8 coordinating with platinum(II) centres to give a triangular metallocycle 9.

Photodynamic therapy (PDT) uses photosensitizers in conjunction with light to generate reactive oxygen species which oxidize and damage tumor cells. PDT is viewed as being less invasive and more targeted than some other anticancer therapies.^[^ ^74^ ^]^ Incorporation of platinum and ruthenium in PDT photosensitizers has the added benefit of the cytotoxicity these metals display, allowing them to also act as chemotherapy drugs in conjunction with PDT.^[^ ^75^ ^]^ Song, Liu, and coworkers designed a novel organic photosensitizing ligand, 8, and incorporated it into a triangular metallocycle with Pt(II) vertices (**Figure** **9**). The resulting metallocycle, 9, was then used to form nanoparticles and their use as anticancer agents was evaluated in vitro.^[^ ^76^ ^]^ The photophysical properties of the ligand and metallocycle were assessed in degassed ethanol solution; upon excitation at 508 nm, 8 was found to be highly fluorescent, with *λ*_PL_ at 569 nm, Φ_PL_ of 78%, and *τ*_PL_ of 8.65 ns. Even in aerated solution, the emission remains strong with Φ_PL_ = 60% and *τ*_PL_ = 8.30 ns. The high Φ_PL_ was largely preserved upon metallocycle formation at 69%. There is an accompanying blue‐shift of the emission to 550 nm and a double‐exponential lifetime with an average *τ*_PL_ = 8.65 ns. Transient absorption spectroscopy studies (*λ*_exc_ at 532 nm) revealed lifetimes of 1.87 and 1.76 μs for 8 and 9, respectively, suggesting that these species could generate reactive oxygen species. The Δ*E*_ST_ value of 8, measured in 2‐methyltetrahydrofuran, was determined experimentally to be 0.085 eV, prompting a temperature‐dependant emission study of the organic ligand, which revealed that the emission intensity for 8 increases with temperature, suggesting a TADF mechanism. This same effect was seen for 9, which suggests that this TADF property is preserved upon complexation, which the authors propose is the source of the relatively long luminescence lifetime of the emitters. The ability of 8 and 9 to generate singlet oxygen was assessed in ethanol solution versus a TPPS standard (TPPS = *meso*‐tetrakis(*p*‐sulfonato‐phenyl) and both displayed high efficiency for oxygen generation with quantum yields of Φ = 95% and 86%, respectively. It is suggested that the small Δ*E*_ST_ value increases the rate of ISC to the triplet state; this in turn increases the rate of ^1^O_2_ production. For in vitro studies, 30 nm nanoparticles of 9 were prepared via nanoprecipitation. These nanoparticles were stable for 4 days in a culture medium, and in blood no significant hemolysis was seen; both were promising results for the biocompatibility of these species. The uptake of 9 nanoparticles by HeLa cells was confirmed by confocal laser scanning microscopy with fluorescent dying of the cell nuclei (cells dyed with 4′,6‐diamidino‐2‐phenyl‐indole = DAPI). Strong fluorescence was seen from the HeLa cells after 12 h, with the green emission of the nanoparticles overlapping with the blue emission from DAPI, confirming uptake of the nanoparticles into the nuclear region of HeLa cells. The accumulation of the nanoparticles within the HeLa cells is attributed to the positive nanoparticles being attracted to the negatively charged cell membrane.^[^ ^77^ ^]^ Singlet‐oxygen generation by the nanoparticle containing HeLa cells upon irradiation was confirmed using singlet‐oxygen sensor green. The half‐maximal inhibitory concentration (IC_50_) of 8 when irradiated in cells was 1.0 μg mL^−1^, and for 9 nanoparticles it was 0.5 μg mL^−1^, which is lower than other PDT agents containing BODIPY dyes and porphyrins.^[^ ^77^, ^78^ ^]^ The greater toxicity of the metallocycle nanoparticles is due to the strong binding interactions of the Pt(II) centers with DNA. The combination index of the PDT/chemotherapy combination offered by the nanoparticles, a measure of how synergistic the two approaches are, was calculated to be 0.13. This value indicates that there is good synergy between the PDT offered by the 8 ligands and chemotherapy effects of the Pt(II) centers.

### 2.7 Confining Carbon Dots within Zeolites to Switch on TADF

**Figure 10:**
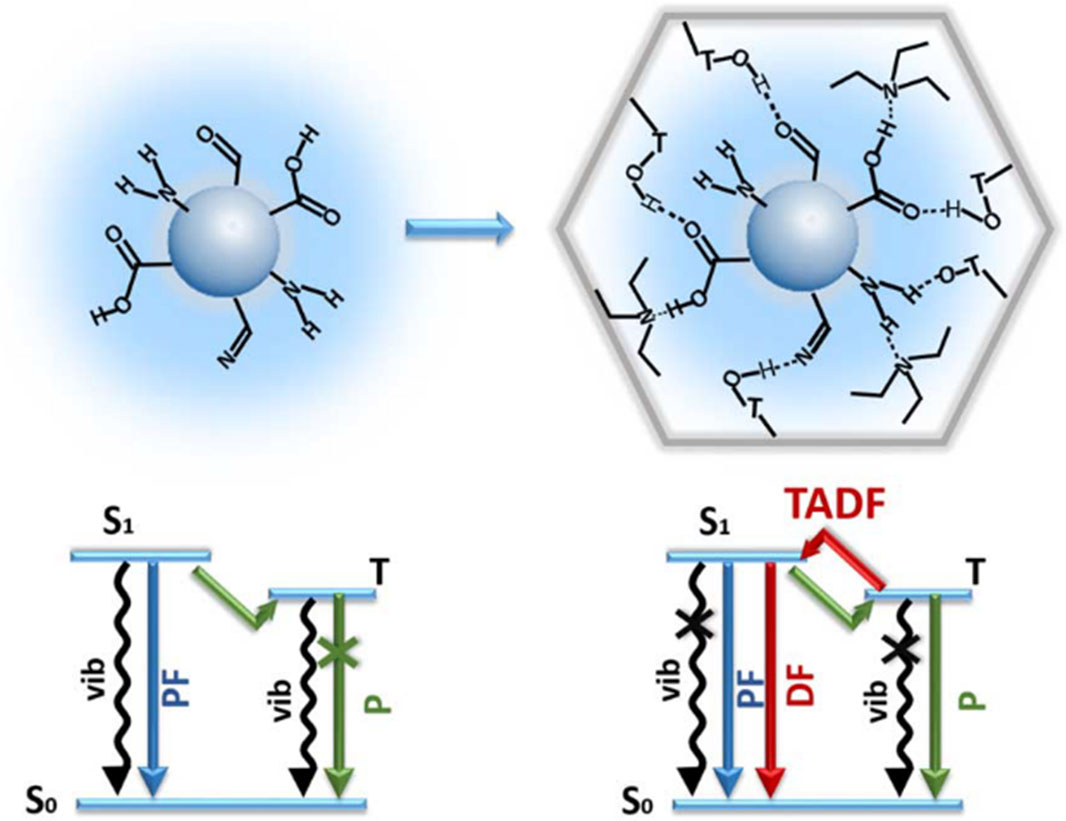
(left) Nonradiative deactivation of discrete carbon dots triplet states following ISC via vibrations and interactions in solution. (right) Switching on of TADF in carbon dots following encapsulation within zeolite frameworks, preventing nonradiative deactivation and allowing rISC to occur. Reproduced with permission.^[^ ^85^ ^]^ Copyright 2017, American Association for the Advancement of Science.

Since their discovery in 2004, carbon quantum dots have gained significant interest due to their remarkable photoluminescence and electrochemical properties combined with their low cost and toxicity/environmental impact.^[^ ^79^, ^80^, ^81^, ^82^, ^83^ ^]^ Emissive carbon dots comprise a carbon nanoparticle core, whose surface is passivated with organic ligands, with the choice of surface groups being key to the emissive nature of these materials.^[^ ^84^ ^]^ Yu and Li demonstrated that the confinement of carbon dots within zeolites allowed these materials to display efficient and long‐life TADF emission (**Figure** **10**).^[^ ^85^ ^]^ Three systems (CD1‐3) were synthesized via solvothermal conditions, forming carbon dots in situ as the zeolite crystallized, which then resulted in embedded carbon dots within the zeolite structure framework. CD1 used triethylamine (TEA) as the structure‐directing agent (SDA) in combination with aluminum tri*iso*propoxide, phosphoric acid, triethylene glycol, and hydrofluoric acid to give hexagonal crystals, with carbon dots of diameter 3.7 nm monodispersed within the structure as confirmed by transmission electron microscopy imaging. CD2 was prepared similarly but with 4,7,10‐trioxa‐1‐13‐tridecanediamine as the SDA and no hydrofluoric acid. Here the dispersed carbon dots possessed an average diameter of 3.5 nm. CD3 was prepared similarly to CD2 but with the addition of dimagnesium phosphate, giving dispersed carbon dots of an average diameter of 3.4 nm. Following excitation at 370 nm, the three structures show deep‐blue emission (C1: 430 nm, Φ_PL_ = 15%, CIE (0.17,0.13); C2: 440 nm, Φ_PL_ = 52%, CIE (0.17,0.14); C3: 425 nm, Φ_PL_ = 23%, (0.17,0.13)). The larger photoluminescence quantum yields observed for CD2 and CD3 compared with CD1 were attributed to the use of 4,7,10‐trioxa‐1‐13‐tridecanediamine as the SDA, which was shown previously to passivate the surface of carbon dots, improving photostability and enhancing photoluminescence quantum yields.^[^ ^86^, ^87^ ^]^ All three systems displayed delayed emission with lifetimes on the order of milliseconds (CD1; *τ*_PL_ = 350 ms, CD2; *τ*_PL_ = 197 ms, CD3; *τ*_PL_ = 216 ms). The TADF character of these systems was confirmed by temperature‐dependent transient photoluminescence decay measurements that showed enhancement of the delayed emission with increasing temperature. The Δ*E*_ST_ values for the three complexes were measured and found to be Δ*E*_ST_ = 0.22 eV for CD1, Δ*E*_ST_ = 0.23 eV for CD2, and Δ*E*_ST_ = 0.22 eV for CD3. The emergence of TADF following the confinement of the carbon dots within the zeolite matrices was attributed from *n* to π* transitions in carbonyl and imine bonds within the carbon dots. These transitions associated with triplet states are of different orbital types than those associated with the CT singlet state. This leads to greater spin−orbit coupling that facilitates rISC. The emission from these systems remains strong in air as the zeolite matrix impedes oxygen from quenching the triplet states of the embedded carbon dots.

**Figure 11:**
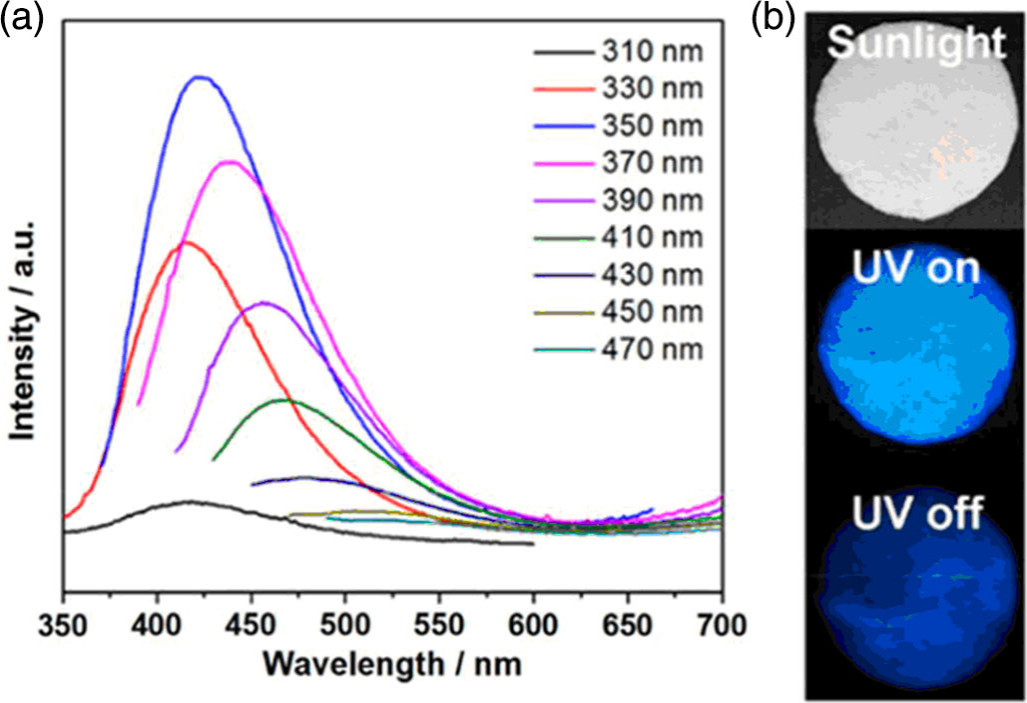
a) Excitation‐dependent emission from CD4. b) Emission from the CD4 is visible after switching off the excitation source, demonstrating long‐life emission. a,b) Reproduced with permission.^[^ ^88^ ^]^ Copyright 2019, American Chemical Society.

Yu and Li went on to report the synthesis of zeolite‐confined carbon dots, which, depending on the template used in the synthesis of the CDs, showed either RTP or TADF.^[^ ^88^ ^]^ The TADF zeolite‐confined carbon dot, CD4, was prepared using a 4,7,10‐trioxa‐1‐13‐tridecanediamine template. Upon excitation at 370 nm, CD4 showed emission at *λ*_PL_ = 440 nm, with an associated quantum yield of Φ_PL_ = 29%. The photoluminescence from CD4 showed some excitation dependence, with the strongest emission seen upon excitation at 350 nm (**Figure** **11**). The authors assigned TADF to this material based on the long‐observed delayed lifetime (*τ*_PL_ = 153 ms) at room temperature under air atmosphere, which was confirmed by temperature‐dependant transient photoluminescence decay measurements, which showed an increase in the delayed emission component with increasing temperature (no prompt lifetime quoted). The Δ*E*_ST_ was measured to be 0.18 eV, which is compatible with rISC at room temperature. The Δ*E*_ST_ of the unconfined carbon dot was slightly larger at 0.21 eV in the mother liquid; however, no long‐life emission is observed at room temperature. The authors attribute the switching on of TADF upon encapsulation of the carbon dot to be due to both the suppression of nonradiative decay pathways and stabilization of the triplet state by the zeolite host by suppressing nonradiative decay processes such as molecular vibrations and rotations.

**Figure 12:**
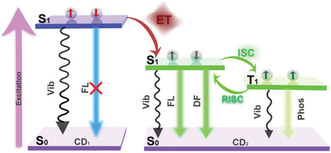
Harvesting of singlet states of encapsulated 4,7,10‐trioxa‐1‐13‐tridecanediamine carbon dots (here labeled as CD_1_) by *m*‐phenylenediamine carbon dots (here labeled as CD_2_), leading to TADF emission from the latter. Reproduced with permission.^[^ ^84^ ^]^ Copyright 2019, Royal Society of Chemistry.

More recently, Yu and Li reported the encapsulation of two different carbon dots within the same zeolite matrix, whereby varying the ratio of the two within the framework allowed for the tuning of the emission wavelength.^[^ ^84^ ^]^ The ratio of the two carbon dot precursors, *m*‐phenylenediamine and 4,7,10‐trioxa‐1‐13‐tridecanediamine, was varied from 0, 0.007, 0.0014, and 0.042 to give four different compositions of carbon dots encapsulated within zeolites, CD5‐8, respectively. Scanning electron microscopy revealed similar polyhedral morphology for all composites, whereas transmission electron microscopy showed good dispersion of the carbon dots within the frameworks. Increasing the ratio of *m*‐phenylenediamine to 4,7,10‐trioxa‐1‐13‐tridecanediamine led to a red‐shifting of the emission (CD5‐8; *λ*_PL_ = 425, 484, 498, 515 nm, respectively). All four composites displayed very long‐life delayed fluorescence, with photoluminescence quantum yields and delayed lifetimes increasing but prompt lifetime decreasing as the *m*‐phenylenediamine content increased (CD5: *τ*_PL_ = 24.43 ns, 271 ms, CD6: *τ*_PL_ = 37.54 ns, and 578 ms, CD7: *τ*_PL_ = 11.76 ns and 801 ms, CD8: *τ*_PL_ = 7.70 ns and 860 ms. CD5‐8: Φ_PL_ = 20.9%, 25.1%, 37.1%, 42.0%, respectively). Temperature‐dependant time‐resolved emission studies of the composites revealed the expected thermal activation of the delayed emission, confirming a TADF mechanism. The Δ*E*_ST_ values for CD5 and CD8 are 0.20 and 0.14 eV, respectively. A mechanism was proposed by the authors, as shown in **Figure** **12**, whereby within systems CD6, CD7, and CD8 Förster resonance energy transfer (FRET) from the 4,7,10‐trioxa‐1‐13‐tridecanediamine carbon dots to the *m*‐phenylenediamine carbon dots within the zeolite framework occurs, which then emit via a TADF mechanism. The FRET efficiency increases as the ratio of *m*‐phenylenediamine to 4,7,10‐trioxa‐1‐13‐tridecanediamine increases.

### 2.8 Organic Macrocycles and Cages

TADF materials commonly comprise twisted donor−acceptor species, with the separation of frontier molecular orbitals leading to a reduced exchange integral and consequently a small Δ*E*_ST_ that results in efficient rISC. Huang and coworkers reported the synthesis of two supramolecular donor−acceptor‐based structures, which enforce highly twisted conformations, decreasing Δ*E*_ST,_ while also providing an increasing rigidity to the emitter moieties that result in narrow emission.

**Figure 13:**
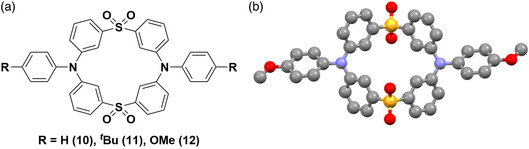
a) Structure of 10, 11, and 12. b) Ball‐and‐cap model of the crystal structure of 12 with sulfur atoms in yellow, oxygen in red, nitrogen in blue, and carbon in gray, with hydrogen omitted for clarity. Crystal structures are reproduced with permission.^[^ ^89^ ^]^ Copyright 2018, Royal Society of Chemistry.

Huang and coworkers reported the one‐pot synthesis of a family of cyclic organic species showing deep‐blue TADF in doped thin films and room‐temperature phosphorescence (RTP) as a crystalline powder.^[^ ^89^ ^]^ The cyclic species is made up of two triphenylamines linked by two sulfonyl groups. The donor groups may be substituted with a *tert*‐butyl or methoxy group to allow tuning of the emission (10 = H, 11 = ^*t*^Bu, 12 = OMe, **Figure** **13**). DFT calculations on these systems showed the LUMO to be largely distributed over the diphenyl sulfone acceptors and the HOMO localized over the triphenylamine donors. The three emitters showed photoluminescence in DCM at 427, 443, and 469 for 10, 11, and 12, respectively, with measured Δ*E*_ST_ values of 0.34, 0.31, and 0.24 eV, respectively. The observed red‐shift of the emission corresponds with the progressive strengthening of the donor, which also leads to a destabilized HOMO. Transient photoluminescence decays of doped thin films [5 wt% mCP (mCP = 1,3‐bis(carbazol‐9‐yl)] of 10, 11, and 12 all showed prompt (3.78 ns, 5.55 ns, and 9.97 ns) and delayed (10.9, 16.1, 101.3 μs) fluorescence under inert atmosphere. The delayed contribution to fluorescence increases with increasing electron‐donating ability of the donor (10 = 10.5%, 11 = 22%, 12 = 80%), attributed to greater localization of the HOMO on the donor, leading to decrease in the Δ*E*_ST_. The photoluminescence quantum yields of the emitters are between 8% and 14% under N_2_. Interestingly, emitters 11 and 12 also display RTP in the crystalline powder, with lifetimes of 4.55 and 5.09 ms, respectively. The origin of the RTP was rationalized by the authors using TD‐DFT calculations to intermolecular interactions between adjacent donor and acceptor units.^[^ ^90^ ^]^

**Figure 14:**
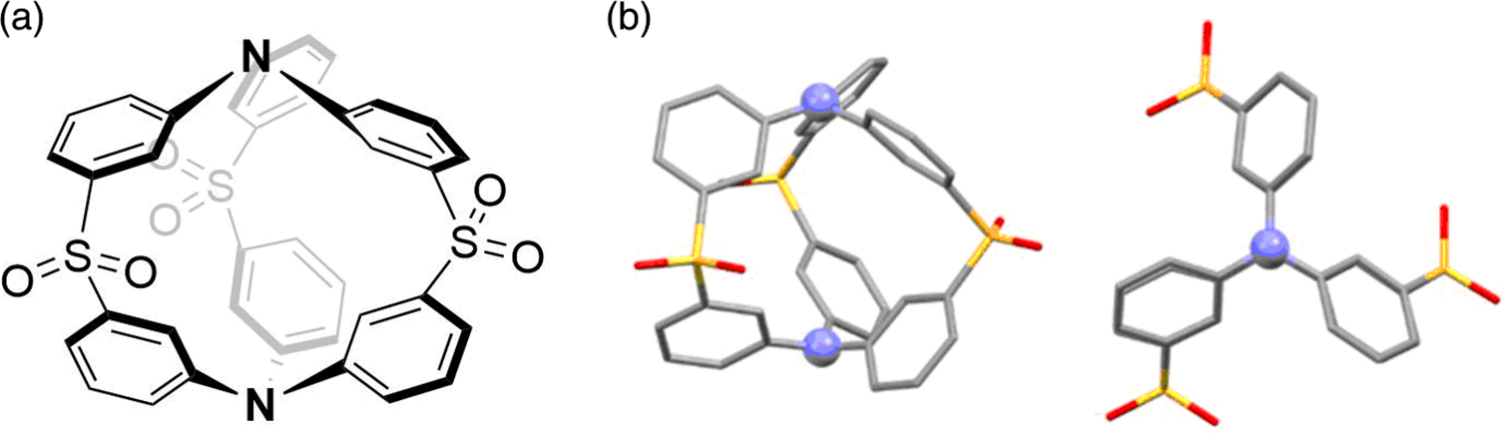
a) Structure of 13. b) Crystal structure of the organic cage 13 from the side (left) and above (right) with sulfur atoms in yellow, oxygen in red, nitrogen in blue, and carbon in gray, with hydrogen omitted for clarity. Crystal structures for (b) reproduced with permission.^[^ ^91^ ^]^ Copyright 2020, Springer Nature.

The same group later reported the one‐pot synthesis of an organic cage that displayed TADF, 13.^[^ ^91^ ^]^ The structure of 13 was unambiguously determined by X‐ray crystallography, having *C*_3h_ symmetry with two triphenylamine molecules linked by three sulfonyl groups, in an analogous manner to the cyclic compound discussed earlier. This structure places the donor triphenylamines next to the acceptor diphenylsulfone units in a twisted fashion (**Figure** **14**). This system showed narrow (full width at half maximum: 34 nm/0.24 eV) deep‐blue emission centered at 414 nm with a small Stokes shift of 43 nm, both linked to the rigidity of the cage. The measured Δ*E*_ST_ of 13 is 0.18 eV, which is significantly smaller than the macrocycles 10‐12. The photoluminescence quantum yield of 13 is only 15%. The time‐resolved transient photoluminescence decay shows both prompt (*τ*_PL_ = 8.65 ns) and delayed (*τ*_PL_ = 8.6 μs) fluorescence at room temperature, with the delayed component increasing in proportion at elevated temperatures, confirming TADF. OLED devices fabricated of 13 showed a maximum external quantum efficiency (EQE_max_) of 2.63%. The electroluminescence spectrum, peaking at 413 nm, retains a narrow FWHM (35 nm/0.25 eV).

### 2.9 Organic Dots

**Figure 15:**
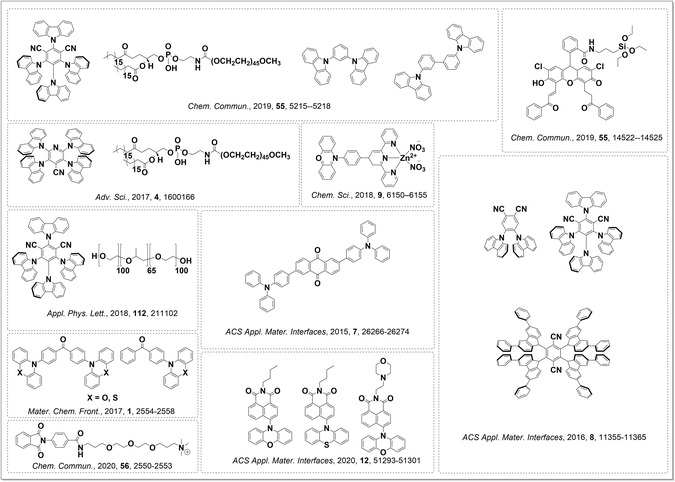
Emitters, solubilizing chains, and host materials that have been used in the construction of TADF‐emissive organic dots.

Organic dots (also referred to as organic nanoparticles) are systems comprising hydrophobic organic cores, decorated with hydrophilic chains, which allow for high water solubility. Over the past few years, multiple groups have reported examples of such micelle‐like species that are emissive via a TADF mechanism. There are multiple examples of 4CzIPN and structurally similar emitters,^[^ ^9^, ^92^, ^93^, ^94^, ^95^ ^]^ as well as phenoxazine derivatives,^[^ ^14^, ^96^, ^97^ ^]^ being used as the organic cores, along with other TADF emitters, which may be decorated with hydrophilic chains, as shown in **Figure** **15**. The hydrophilic chains can be covalently linked to the organic emitter or the two components may be suspended in solution and mixed to self‐assemble the separate components into a micelle. The hydrophilic chains used vary, with a popular choice being derivatives of polyethylene glycol, boasting a number of benefits including low cost, low toxicity, and excellent water solubility.^[^ ^9^, ^92^, ^93^, ^94^, ^98^ ^]^ An example from Tang and Zhao shows the use of peptide chains to facilitate passage through cell and nuclear membranes,^[^ ^96^ ^]^ which illustrates the main application of these TADF organic dots as emissive biological probes. The use of hydrophilic chains is not essential, with examples where nanoparticles/nanorods formed due to aggregation of organic emitters in aqueous media^[^ ^95^, ^99^ ^]^ or the use of Zn(II) ions to initiate a complexation of an organic species to give a water‐soluble complex.^[^ ^97^ ^]^ Biological autofluorescence makes it hard to distinguish emission from the probe from background cell emission when the excited state of the probe is too short‐lived. The use of phosphorescent complexes that possess long‐life triplet excited states is somewhat problematic due to the toxicity of the heavy metal complexes. TADF provides an answer to this problem, being both long‐lived and not as toxic as heavy metal complexes; however, the presence of oxygen in biological systems quenches triplet states and can lead to a reduction in photoluminescence quantum yield. Shielding of the TADF emitters within organic dots allows for high photoluminescence quantum yields under ambient atmosphere in solution that are insensitive to the presence of oxygen. The wide range of emitters and, where used, water‐solubilizing groups, demonstrates the tunability and versatility of this class of supramolecular materials.

### 2.10 Self‐Assembled Fibers

**Figure 16:**
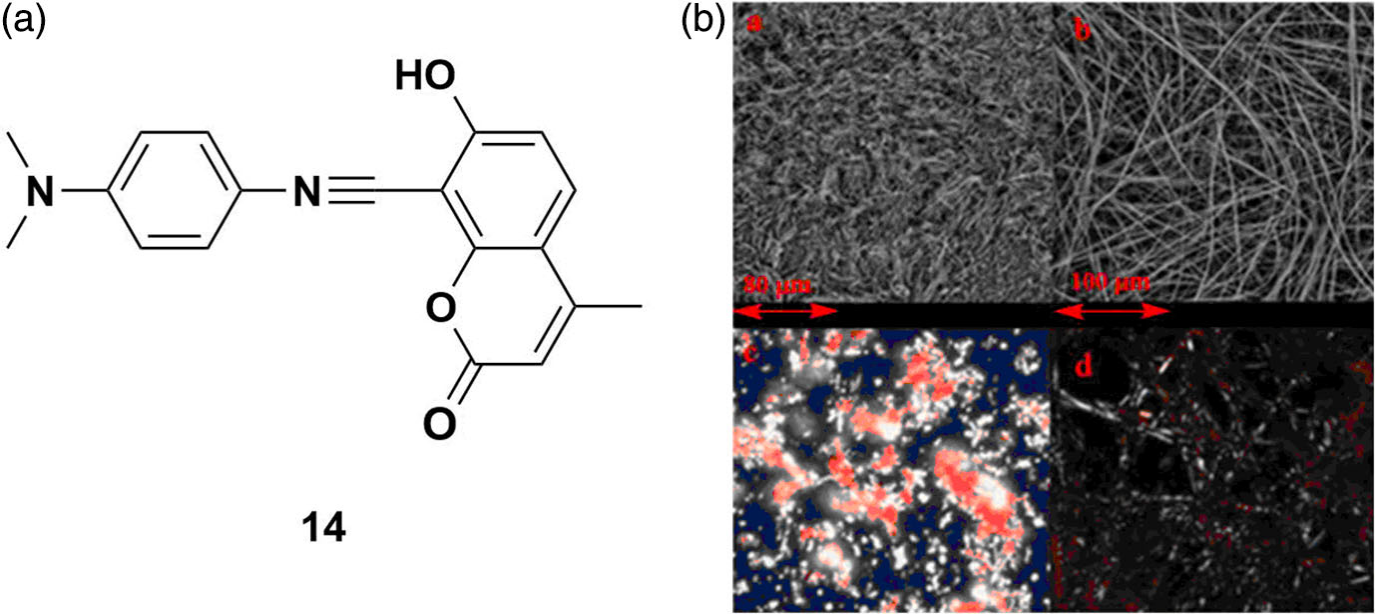
a) Structure of emitter 14. b) SEM of powder (top left) and nanostructure film (top right) and POM images of the powder (bottom left) and self‐assembled nanofibers (bottom right). b) Adapted with permission.^[^ ^100^ ^]^ Copyright 2016, Royal Society of Chemistry.

Qi et al. reported a novel TADF emitter, 14, that displays AIE and can self‐assemble into microfibers (**Figure** **16**).^[^ ^100^ ^]^ Increasing the water fraction beyond 40% of a DMF solution results in an increase in the emission intensity attributed to aggregation of 14 as it becomes less soluble in solution. By varying the water content, the size of the aggregates can be adjusted, from 20 nm in 40% water content up to 10.0 μm in 90% water content solution. The self‐assembly of 14 into fibrils was achieved by slowly evaporating a THF:CH_2_Cl_2_ (1:1 v/v) solution of 14 on a quartz substrate. Differences in the photophysical properties can be seen between the powder and thin film, with a small red‐shift in the emission from 640 to 645 nm and an increase in the photoluminescence quantum yield from 13% to 26%. Scanning electron microscopy (SEM) and polarizing optical microscopy (POM) showed that the powder formed needle‐like crystals, whereas the thin film contained microfibers that were up to several hundred micrometers long and several hundred nanometers wide (Figure 16b). The TADF properties of the thin film were confirmed by transient photoluminescence decay measurements that showed the presence of prompt (2.3 ns) and delayed (10.0 μs) fluorescence and temperature dependence of the delayed component of the emission. The Δ*E*_ST_ of free 14 was calculated via DFT to be 0.61 eV, which, despite the large energy difference, is of similar magnitude to that of a previously reported TADF compound.^[^ ^101^ ^]^

## 3 Conclusion

We have presented a diverse set of examples of supramolecular systems that exhibit TADF. This area, however, remains rather underexplored, and the potential of these systems is not yet fully realized. What is clear is that the photophysical properties can be finely tuned within the self‐assembly. This sometimes leads to new reactivity, to a loss of TADF, or in other examples, to a turn‐on of the TADF mechanism. We hope that as the chemical space associated with compounds that exhibit TADF continues to expand, so too will related supramolecular systems. The examples presented in this mini‐Review demonstrate the potential of supramolecular TADF systems and we hope that this will spur further development in field.

## Conflict of Interest

The authors declare no conflict of interest.
